# The importance of time and other determinants in the assessment of heavy metals release during solid waste management

**DOI:** 10.1038/s41598-023-28926-0

**Published:** 2023-01-30

**Authors:** Kamila Mizerna, Anna Król

**Affiliations:** Faculty of Mechanical Engineering, Opole University of Technology, Mikołajczyka Str. 5, 45-271 Opole, Poland

**Keywords:** Environmental chemistry, Environmental impact

## Abstract

One of the parameters affecting the leachability of heavy metals from waste is their contact time with the leachant. In this paper, the leaching behaviour of Zn, Cu, Pb and Ni was evaluated in relation to the liquid to solid ratio (L/S), which is a reflection of time after which a certain volume of water permeates the material, e.g. in slag heaps or landfills. A leaching study was carried out by different leaching methods with using three test materials, i.e. hazardous zinc slag, lump copper slag and mineral-organic composite. It was found that the highest amount of metals leached in the long term in the maximum availability test, under the following leaching conditions: L/S = 50 dm^3^/kg, reduced pH of the leachant, fragmentation of the materials to particle size < 0.125 mm. Comparing the results obtained in the batch test and the percolation test, no strict trend was observed in the release of a given metal from different test materials. The analysis using the tank test showed that processes controlling leachability can result in the release of the highest metal loads immediately after contact between the material and the leachant, but can also contribute to the release of metals only after prolonged contact.

## Introduction

The issue of the leachability of heavy metals from different materials and products is the key to environmental impact assessment. Heavy metals migrate form products made of natural raw materials, waste materials and from waste deposited in the environment^1–3^.

A major concern in evaluating the potential release of elements from waste materials deposited in landfills is that with the passage of time, unacceptable amounts of contaminants may be released into the environment^4^. Time is an important factor affecting the concentrations of leached contaminants when evaluating the rate at which landfill processes take place. These processes may limit the release of elements when the reaction is slow (slow dissolution of minerals) or during diffusion. As waste is stored over time, changes in its properties and environmental conditions may occur due to the decomposition of organic matter, changes in the permeability of the landfill, the carbonation of alkaline waste materials, or the increase in the leaching surface area due to erosion^5^.

Laboratory leaching tests can include single batch tests/extractions and multiple extractions/dynamic tests. One feature common to all leachability tests is the generation of an eluate that is then used to evaluate specific material properties or to simulate leaching scenarios in the environment^6^. One of the criteria for the division of leaching methods is the duration of leaching, so we can distinguish between long-term tests (e.g. tank test^7^) and short-term tests (e.g. batch test^8^) and short-term tests to predict the long-term leaching (maximum availability test^9^).

The ratio of the mass of the solid to the volume of the leachant affects the rate of leaching of heavy metals from waste^10–13^. Expressing leachability test results in terms of leached metal amount (in mg/kg) is a convenient way. It allows comparing results obtained from different test methods, primarily from percolation^14^ and batch tests^8,15,16^. However, it should be kept in mind that the L/S ratio in a leachability test can also be affected by pH or other parameters controlling leaching. In addition, different test conditions such as sample shaking, temperature, type of leachant, and contact time may vary, making it difficult to compare the results obtained by various test methods^17^.

The leachability of heavy metals is usually assessed by accelerated tests. Short-term leaching tests are often batch tests at a low liquid-to-solid ratio, i.e. L/S = 2 dm^3^/kg corresponding to an environmental (landfill, slag heap) leaching period of 100 years^18^. In most cases, such tests provide satisfactory preliminary assessments of contaminant leaching. Short-term tests can also be performed using column or lysimeter tests. These tests are more accurate, but are conducted over longer periods of time (requiring months or even years) and are therefore not practical for routine estimation of long-term leaching until the L/S ratio reaches the figure of several hundred^4^. The long-term leachability analysis presented by Astrup et al.^4^ shows the prospect of release of elements including heavy metals until L/S = 5000 dm^3^/kg is reached, which corresponds to about 100,000–250,000 years of waste disposal.

Evaluation of long-term leaching, above L/S = 2–10 dm^3^/kg, is generally more complicated. Batch tests provide information on the average leaching expected at the waste landfill site until a fixed L/S ratio is reached in the test. This means that although the total amount of constituents released in the batch test at L/S = 100 dm^3^/kg may reflect the accumulated flow from the landfill site at the same L/S, the composition of the leachant does not reflect the elaute from the site at L/S = 100 dm^3^/kg. Therefore, long-term leaching tests are most often characterized in terms of the amount of element available for leaching, e.g., maximum availability leaching test^9^. Such tests aim to determine the fraction of components that can be released under extreme conditions, i.e. at low pH and after a very long time of landfilling in the environment^4^.

The purpose of the paper is to evaluate the short and long-term leaching effects under the influence of different environmental factors. The study provides the knowledge about leaching behaviour of zinc, copper, lead and nickel from mineral wastes (metallurgical slags) as well as from mineral-organic material (composite). The tested materials were characterized by a different hazard degree to the environment. The selected heavy metals were chosen due to the fact that they belong to the basic group of metals whose content is determined in all components of the environment. Cu, Pb and Ni are considered as the main anthropogenic environmental contaminants^19^. In addition, Zn, Cu, Pb and Ni are present in the composition of test materials and are released from them in contact with water. The process of heavy metal release was observed under laboratory conditions while conducting four leachability tests to simulate different leaching scenario (long-term and short-term). The importance of the research is that the article shows not only the effect of time, but also other parameters affecting leaching (pH, liquid to solid ratio, waste form). The research results are important because of the presentation of comprehensive approach to assessing the impact of waste on the environment. The authors indicate that the use of one leaching method is insufficient in assessing the risk of depositing waste in the environment. Although this practice is common in Europe, compared to the research presented in the article, it is not correct to determine the actual level of leaching of heavy metals in the environment.

## Materials and methods

### Sampling and material preparation

Changes in the leaching level of heavy metals (Zn, Cu, Pb and Ni) over time were analyzed using three test materials. Two of them were metallurgical slags: zinc slag (ZS) and lump copper slag (LCS). The third test material was mineral-organic composite (MOC). The criterion for the selection of waste materials was their different management direction (on landfills and slag heaps or their spreading on the surface of the ground for reclamation purposes).

ZS was generated in the rotary kiln of the zinc smelter in the lead refining section. The slag in the smelter was classified as a waste deposited in the hazardous waste landfill (according to^20^). The samples were collected from the company’s landfill from the slag deposition sector. The material was characterized by a dark grey colour, with ceramic inclusions as well as metallic inclusions of lead grains. ZS was characterized by the different grain size (< 0.1–10 mm). The samples in the form of lumps of irregular shape were also collected.

LCS was obtained in the process of smelting briquetted copper concentrates in a shaft kiln. This waste was classified as inert^20^. The slag is temporarily deposited in heaps because it is used in road construction and cement industry. LCS extracted for the research was in the lump forms of irregular shapes and sizes.

MOC was a mixture of stabilized municipal sewage sludge and ashes from household solid fuel combustion. The material was classified as a waste deposited in the nonhazardous landfill^20^ and used in landfill reclamation. The material was characterized by a granular structure similar to that of light soils.

All test material samples were collected in Poland in accordance with EN 14899:2005^21^. Representative laboratory samples were prepared by grinding to the particle size < 4 mm (batch test and percolation test), < 0.125 mm (maximum availability test). Lumps of known surface area and mass to the tank test were also prepared.

The test materials had physical properties listed in Table 1.

**Table 1:** Physical properties of test materials.

Material	Sample name	Humidity (%)	Density (Mg/m^3^)	Porosity (%)
Zinc slag	ZS	8.9	4.28	53.7
Lump copper slag	LCS	11.2	2.15	17.0
Mineral-organic composite	MOC	21.5	0.84	74.2

### Leaching methods

#### Batch test

The test of heavy metals leaching from waste materials was performed according to the procedure based on the standard EN 12457-2:2002^8^. The required grain size of samples was < 4 mm. Water extracts were prepared at liquid/solid ratio of L/S = 10 dm^3^/kg. As the leachant was used a deionized water with the electrical conductivity ≤ 10 µS/cm. Water extracts were shaken for 24 h.

#### Maximum availability test

Maximum availability leaching test was conducted according to EA NEN 7371:2004^9^ for samples of grain size < 0.125 mm. Availability is defined as the potential upper limit of the element that can be released from a solid material into the aquatic environment^25^. The test is a two-stage test until the eluate pH reaches 7 in the first stage and pH 4 in the second stage, with a liquid to solid ratio of L/S = 50 dm^3^/kg. The study was carried out using a TiroLine 7000 titration apparatus from SI Analytics. The material was mixed with water by means of a magnetic stirrer for 6 h in total. 0.2 mol/dm^3^ HNO_3_ for ZS and MOC and 1 mol/dm^3^ HNO_3_ for LCS were used for the study. Since the initial pH value of the eluate for ZHC slag was below 7, the waste was leached without adding a reagent (material's own pH) for 3 h in the first stage. The eluates from the two stages were filtered and combined to evaluate heavy metal concentrations.

#### Percolation test

Evaluation of heavy metal release during percolation process was carried out based on column test according to EN 14405:2017^14^ for samples of grain size < 4 mm. A column with a diameter of 5 cm and a filling height of 30 cm was used in the test. The empty column was weighed and then the test portion was filled in the column. The volume of filled material in the column was 0.6 dm^3^. The waste column was also weighted and deionized water (with conductivity ≤ 10 µS/cm) percolating up the column was supplied using a peristaltic pump. The water flow rate was 12 ml/h. The column was filled with water until all the material was saturated. It was then left for 3 days to reach equilibrium of the system.

In the next step, 7 eluate fractions were extracted with a fixed cumulative liquid/solid ratio, i.e. L/S = 0.1; 0.2; 0.5; 1; 2; 5; 10 dm^3^/kg based on the guidelines in standard^14^.

#### Tank test

For monolithic waste, a tank test was performed based on the procedure in EA NEN 7375:2004^7^. The purpose of the test was to simulate leaching of metals from the materials as a function of time. The test was conducted using two test materials, i.e. ZS and LCS. Waste pieces were prepared for the test in the form of lumps of known mass, surface area and volume, representative of the test material. According to the standard, the smallest dimension of the piece was greater than 40 mm. Mineral-organic composite (MOC) was not tested in the tank test because of the presence of this material only in shredded form, not meeting the size criterion.

The samples were placed in polyethylene (PEHD) containers, on supports, to be surrounded on all sides by the leachant. According to the standard, the volume of liquid in the container was 2–5 times the volume of the sample. The wastes were subjected to leaching with water of pH 7 (sample designations: ZS pH7 and LCS pH7) and additionally, for comparison purposes, with liquid of pH 4 (ZS pH4 and LCS pH4). A 65% solution of HNO_3_ was used to lower the pH. For the leaching test with liquid at pH 4, for LCS, two pieces of waste were used due to the fact that the smallest piece dimension was less than 40 mm. In this case, the geometric area of the sample was the sum of the areas of the individual pieces. The eluates used in the test were extracted according to the following time schedule: after 0.25; 1; 2.25; 4; 9; 16; 36 and 64 days. After each extraction, the leachant were completely replaced and the samples were immersed in the liquid another time.

Tank test can be used if the matrix of the material does not dissolve. To assess solubility, two main criteria depending on the pH and conductivity of the eluates must be checked. If criterion 1 (Eq. 1) is not met, the matrix is considered not to be soluble. The leachability can then be determined using the tank test. On the other hand, if criterion 1 is met, criterion 2 (Eq. 2) must be checked^7^.

1 $$S_{7 - 8} > 1,5 \cdot \frac{{V_{p} }}{V} + 10^{{{\text{pH}}_{7 - 8} - 11,78}} + 10^{{2,5 - {\text{pH}}_{7 - 8} }}$$

2 $$S_{7 - 8} > 2 \cdot S_{5 - 6}$$

where *S*_7–8_—average value of the measured conductivities in periods 7 and 8 (µS/cm), *S*_5–6_—average value of the measured conductivities in periods 5 and 6 (µS/cm), pH_7–8_—average pH value in periods 7 and 8, *V*—volume of leachant (dm^3^), *V*_*p*_—volume of the test piece (dm^3^).

If criterion 2 is not met, further assess heavy metal leachability can also be carried out.

### Chemical analysis and treatment of eluates

The chemical composition of tested materials was determined according to the standard EN 196-2:2013^22^ by applying the X-ray fluorescence method (XRF) using Axios Cement Panalytical spectrometer. The loss on ignition was designated by the weight method in accordance with EN 15935:2012^23^. For total content assessment of Zn, Cu, Pb and Ni, samples were mineralised in a closed system in accordance with EN 13657:2002^24^. The aqua regia was used as a reagent (65% HNO_3_ and 35% HCl). The process was carried out in closed teflon vessels with the use of microwave mineraliser (Start D, Milestone).

Eluates from leaching tests and mineralizates were prepared in triplicate and filtered through 0.45 µm membrane filters. All eluates were tested for the pH and electrical conductivity value. Then they were fixed by 65% HNO_3_. Analysis of Zn, Cu, Pb and Ni content in eluates and mineralizates were determined by flame atomic absorption spectrometry (FAAS) using Thermo Solaar 6M spectrometer. Each measurement was carried out with three repetitions holding relative standard deviation (RSD) < 5%.

### Quality control

The certified reference materials (CRM) were analyzed after digestion in aqua regia for quality control of total metal content determination in the samples. Two CRM materials were analyzed. The recovery of CRM ‘Metals in soil’ SQC001 (Merck) was as follows: Zn 104%, Cu 106%, Pb 103%, Ni 107%. The recovery of CRM ‘Rock’ NCS DC73303 was as follows: Zn 96%, Cu 94%, Pb 90%, Ni 93%. For each tested parameter, three repetitions were made for all samples. Data were subjected to analysis of basic statistics (mean, SD, min–max, variance) using the Statistica 13 software. Mean values were compared using the test of least significant difference (LSD). Data were analyzed using analysis of variance (ANOVA) to evaluate whether the means were significantly different (significant level for *P* ≤ 0.05).

### Leachibility calculation

The results of Zn, Cu, Pb and Ni concentrations obtained in the batch test, percolation test and maximum availability test were converted to mg/kg dry matter based on Eq. 3^8,14,19^:

3 $$U_{i} = \frac{{c_{i} \cdot V_{i} }}{{m_{0} }}$$

where *U*_*i*_—released quantity of an element per quantity of sample for analysis in eluate fraction *i* (mg/kg d.m.), *V*—volume of the eluate fraction *i* (dm^3^), *c*_*i*_—concentration of the element in the eluate fraction *i* (mg/dm^3^), *m*_0_—dry mass of the test sample (kg).

For the percolation test, the cumulatively released quantity (Σ*U*_*i*_) of heavy metals representing the sum of the released quantity of an element (*U*_*i*_) obtained in all eluate fractions was determined. When metal concentrations were recorded below the limit of quantification, the cumulative leachability was given as a range of values. The lower limit of the interval was calculated by substituting in place of the concentrations below the quantification limit, the values equal to zero, while the upper limit was taken as the quantification limit for the element. The uncertainty of cumulatively released quantity *u*_*c*_ (Σ*Ui*) was calculated as the root of the sum of squares of uncertainties of leaching results in individual fractions of the eluate.

The leachability of heavy metals in tank test was determined according to equation^7^:

4 $$E_{i}^{*} = \frac{{c_{i} \cdot V}}{A}$$

where *E*_i_^*^—measured leaching of a component in fraction *i* (mg/m^2^), *c*_*i*_—concentration of the component in fraction *i* (mg/dm^3^), *V*—volume of the eluate (dm^3^), *A*—surface area of the piece (m^2^).

Measured cumulative leaching ($\varepsilon_{n}^{*}$) of an element was calculated according to the formula^7^:

5 $$\varepsilon_{n}^{*} = \mathop \sum \limits_{i = 1}^{n} E_{i}^{*} \quad {\text{for}}\;{\text{n}} = {1}\;{\text{to}}\;{\text{N}}$$

where $\varepsilon_{n}^{*}$—measured cumulative leaching of an element for period *n* comprising fraction *i* = 1 to *n* (mg/m^2^), $E_{i}^{*}$—measured leaching of the element in fraction *i* (mg/m^2^), *N*—total number of leachant replenishment periods.

## Result and discussion

### Characterization of test materials

Table 2 shows the chemical composition of the materials as oxide forms of the elements. The major components of ZS were Fe_2_O_3_, Al_2_O_3_, and SiO_2_. These three compounds together accounted for over 67% of the waste mass, of which 41.9% was Fe_2_O_3_. The proportion of sulfur (in the form of SO_3_) and alkali (Na_2_O + K_2_O) was determined next. In the chemical composition of LCS and MOC, a high content of silica SiO_2_ was found (42.8% and 37.5%, respectively). For LCS, SO_3_ was determined at the lowest level (0.12%). Negligible or no ignition loss was determined in ZS and LCS slags. As reported by Bożym et al.^26^ and Mu et al.^27^, the loss on ignition indicates a mineral nature of waste. On the other hand, for MOC, ignition losses were determined at high levels. This indicates a presence of organic matter^28,29^.

**Table 2:** Chemical composition of test materials.

Material	Content (wt%)								
	SiO_2_	SO_3_	Al_2_O_3_	Fe_2_O_3_	CaO	MgO	Na_2_O	K_2_O	LOI*
ZS	10.02	7.61	15.33	41.86	2.10	0.98	3.38	0.27	2.68
LCS	42.76	0.12	15.56	17.38	11.88	7.29	1.09	3.38	0.00
MOC	37.50	1.93	2.15	5.57	1.44	4.67	1.10	0.95	18.60

*Loss on ignition.

### Total content and leaching of heavy metals in different methods

The tested wastes showed the presence of analyzed heavy metals (Table 3). The highest content of heavy metals was found in zinc slag (ZS). For this waste the highest level of copper content was determined. It results from technological process of lead refining, where one of the stages is decopperization of lead. Lump copper slag (LCS) and mineral-organic composite (MOC) were characterized by the highest share of zinc.

**Table 3:** Total heavy metals content and their concentration obtained in batch test, maximum availability test (mean ± SD, *n* = 3) and percolation test (mean ± *u*_*c*_(Σ*U*_*i*_), *n* = 7).

Element	Material	Total content (mg/kg d.m.)	Batch test (L/S = 10) (mg/kg d.m.)	Maximum availability test (L/S = 50) (mg/kg d.m.)	Percolation test—cumulatively (Σ*U*_*i*_) (L/S = 10) (mg/kg d.m.)
Zn	ZS	61,076 ± 3877	1111.9 ± 72.4	20,136.8 ± 979.0	3081.0 ± 167.0
	LCS	7833 ± 148	1.0 ± 0.1	81.7 ± 3.6	< 0.3
	MOC	680 ± 9	0.3 ± 0.0	289.3 ± 18.0	(0.1–0.4) ± 0.0
Cu	ZS	101,023 ± 14,199	13.2 ± 0.5	21,937.9 ± 1410.9	24.7 ± 2.5
	LCS	4679 ± 471	0.3 ± 0.1	247.6 ± 19.1	1.5 ± 0.2
	MOC	330 ± 37	3.0 ± 0.2	10.0 ± 1.1	0.7 ± 0.1
Pb	ZS	87,410 ± 5865	35.1 ± 0.8	1426.6 ± 22.0	23.5 ± 1.8
	LCS	2364 ± 117	< 0.5	214.3 ± 13.2	(0.0–0.4) ± 0.0
	MOC	43 ± 6	< 0.5	26.3 ± 2.2	< 0.5
Ni	ZS	2402 ± 187	44.1 ± 3.1	84.1 ± 3.5	56.6 ± 2.8
	LCS	42 ± 15	< 0.1	10.7 ± 0.7	1.2 ± 0.1
	MOC	45 ± 3	0.2 ± 0.0	23.6 ± 1.5	1.8 ± 0.3

The three test methods used allowed the release of heavy metals at very different levels. Much higher leachability of Zn, Cu, Pb and Ni was obtained in the maximum availability test than in the batch test and percolation test. The highest leaching of Zn, Pb and Ni relative to total content in this test was observed for the mineral-organic composite (MOC) at 43%, 61% and 52%, respectively. For comparison, in the batch test for these elements, leachability of Zn was: 0,04%, Pb: 0,0%, Ni: 0,4%, and in the percolation test Zn: 0.009–0.05%, Pb: 0.0%, Ni: 4.1%. Copper was released at the highest level from zinc slag (ZS) at 22% in the maximum availability test. It is worth noting that the conditions of this test also resulted in the release of some metals well above the limit of quantification, and their concentrations were not detected in the batch test or percolation test.

Percolation test is a test conducted for granular waste until L/S = 10 dm^3^/kg, similar to the batch test. Therefore, when comparing the results from both methods with each other, it was observed that the ZS waste had lower leachability of Zn, Cu and Ni in the basic test than in the whole test cycle in the percolation test. In addition, it is worth noting that in the case of LCS waste, the release of lead and nickel was observed under percolation conditions, while in the basic test the leachability of this metal was determined below the limit of quantification. Lower leachability of nickel in the basic test was also observed in the MOC composite, similarly to copper from LCS. A different leaching trend was observed for copper from MOC, which was determined at a much lower level under percolation conditions than in the batch test.

**Figure 1:**
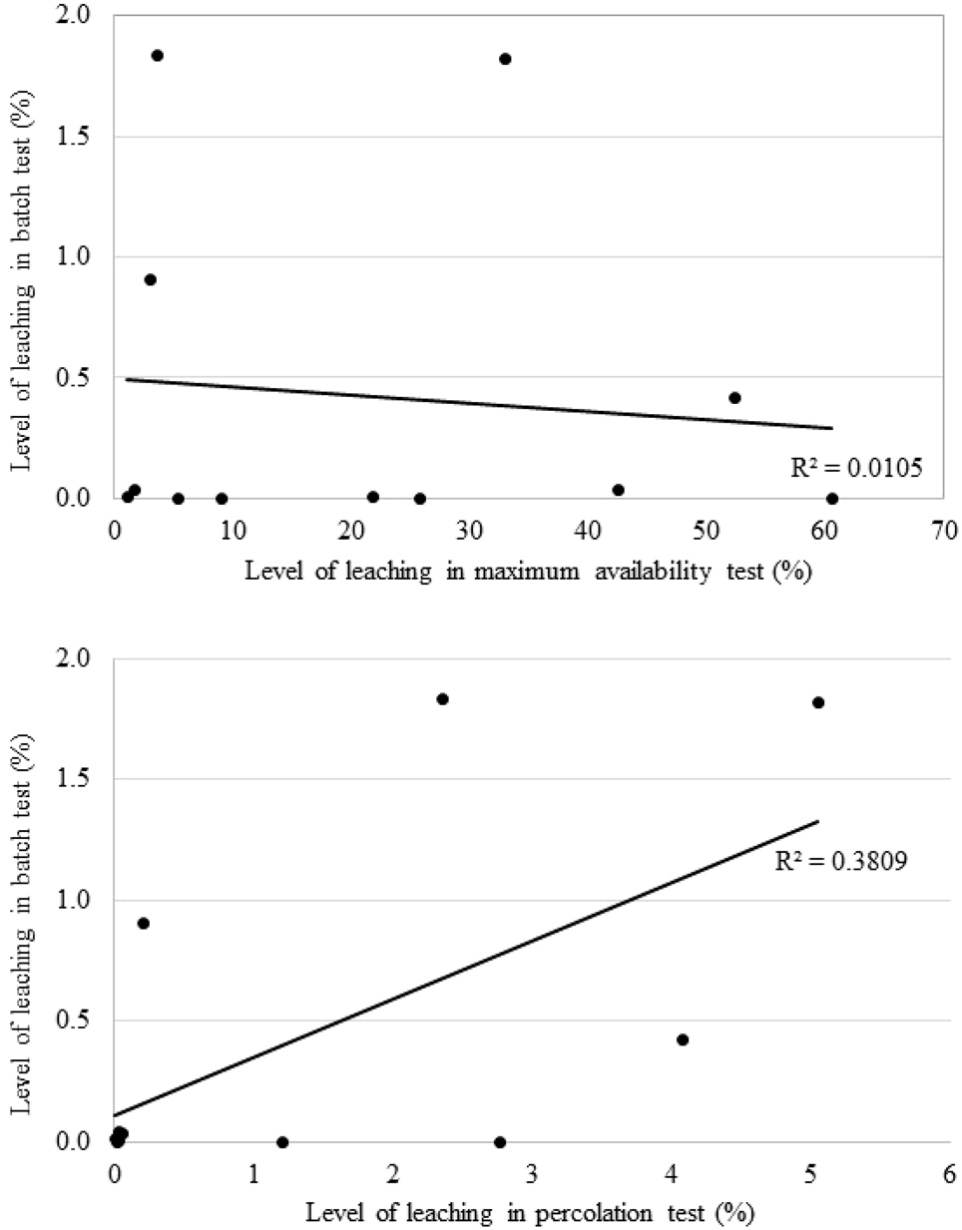
Correlation among the batch test results and maximum availability test as well as percolation test results relative to the total heavy metal contents in materials.

For the results of heavy metals leaching obtained by different test methods, correlations among all the results of heavy metals leaching relative to the total content were determined (Fig. 1). For this purpose, the percentage levels of metals leaching relative to their total content were determined. The compilation of results was for illustrative purposes. The results obtained in the batch test and the maximum availability test were compared in order to compare leachability obtained by two different methods, conducted under different conditions and different L/S ratio, which are, however, short-term methods conducted under static conditions. In turn, the results obtained in the batch test and the percolation test were compared with each other to show the correlation at the same L/S ratio and for the same sample grain size. The leaching results were divided by the total heavy metal contents in waste to normalize the data for different metals and waste samples. The batch test results do not show positive correlation with the maximum availability test results (R^2^ = 0.0105). The batch test and percolation test results in total content shows better correlation with a correlation coefficient value (R^2^) of 0.3809. The comparison of different leaching methods is a practice observed in the literature. Similar comparisons with the determination of the correlation coefficient against various leaching standards were also carried out by Kim et al.^30^ and Yin et al.^31^.

The differences in the level of leaching of waste constituents are due to the conditions under which the leaching process was conducted. It should be noted that conditions such as reduced leachant pH and continuous stirring using a magnetic stirrer increased the level of discharge of mobile forms of heavy metals into the solution. Strong refinement of the waste to a size < 0.125 mm may also have influenced the increased leachability^17,32^. The contact time of the material with the leachant, is also a very important aspect. In the batch test, material samples were subjected to leaching for 24 h, and in the maximum leaching availability test, only for 6 h. However, already such a short time contributed to a much more intense leaching. It cannot, however, be strictly determined which factor may have had a determinant effect on the leaching levels of the heavy metals analyzed. Furthermore, the contact of the test system with atmospheric air could also have had an influence. In the batch test the analysis was carried out in a closed system (limited access of air) and in the maximum leaching availability test in an open system. Hence, the absorption process of CO_2_ from atmospheric air, causing a decrease in the pH of the solution and thus an increase in the solubility of heavy metals, may have contributed to the increased intensity of metal release. A parameter affecting the release of heavy metals, for both methods discussed, is certainly also the liquid/solid L/S ratio used in the study (L/S = 50 dm^3^/kg in EA NEN:7371^9^ and L/S = 10 dm^3^/kg in EN 14405:2017^14^). The L/S ratio reflects the time the waste is subjected to leaching in the environment, landfills, and slag heaps. The maximum availability test shows the scenario of long-term leaching under extreme conditions in an aerobic environment, after decomposition of the material or when the acid neutralizing capacity is lost.

In the case of the percolation test, it is conducted under dynamic conditions, while the basic test is conducted under static conditions. In addition, the factors that regulate the leaching process of contaminants in the column may promote the precipitation of other compounds (salts, oxides), which is also emphasized by Fu and Lu^33^. Consequently, changes in the conditions of the leaching process may cause a decrease in the mobility of some heavy metals, which may have occurred for Cu from composite (MOC) and Zn from lump copper slag (LCS). However, this is not the rule. As shown in the zinc leaching study by Hage and Mulder^34^, this element was released in higher amounts in the percolation test than in the basic test, as was zinc from zinc slag (ZS) and composite (MOC) in the study of the authors of this paper. Thus, a strict relationship in metal leaching behaviour cannot be determined. It is also worth noting that the metal release was certainly affected by the contact time of the material with the leachant. In the batch test this contact is 24 h, while in the percolation test even tens of days, depending on the physical properties of the material. The comparison of the released amounts of heavy metals by the two methods discussed was aimed at providing more information on the behaviour of the leaching of heavy metals from the analyzed wastes at a given L/S ratio. By using the percolation test, more information can be obtained about the release of contaminants because it is possible to analyze the level of leaching with the change in L/S ratio and the duration of the test. Obtaining many different levels of heavy metals leaching at various L/S ratio was also observed by Yao et al.^10^ and Yin et al.^31^.Percolation leachability methods also better approximate the conditions under which waste materials may be present, such as conditions in the aeration zone of landfills. However, they are lengthy and labor-intensive tests, also requiring a sufficiently large mass of test material for a single test.

### Leaching from monolith as a function of time

For the monolithic (integral) test materials, the solubility criteria according to Eqs. 1 and 2 were not met, hence it was concluded that the matrices did not dissolve. Therefore, a heavy metal leachability study was carried out in a tank test^7^, lasting 64 days. Using the test, it was possible to directly evaluate the effect of the contact time of the leaching liquid with the monolithic waste on the leachability of heavy metals.

**Figure 2:**
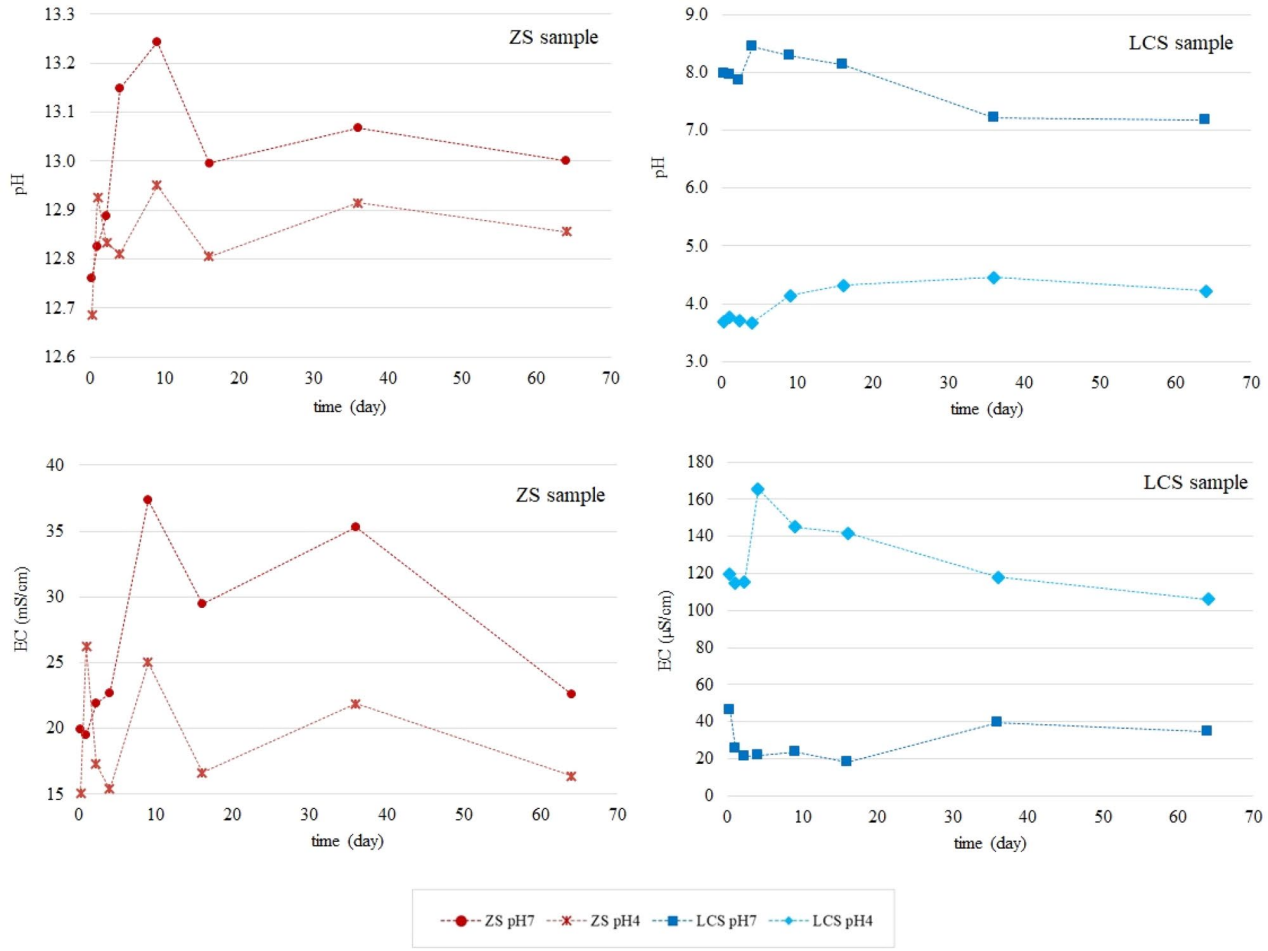
Changes in pH and EC of eluates extracted during 64 days of the tank test.

Figure 2 shows the changes in pH and electrical conductivity (EC) values of the different eluate fractions with respect to the duration of the test. All extracted eluates from ZS waste were alkaline (pH in the range 12.76–13.24 for the ZS pH7 sample and 12.69–12.95 for the ZS pH4 sample). From day 9 of the study onwards, the trends in the pH changes of the eluates were similar for both samples. It should be noted that the use of a pH 4 leachant did not reduce the pH values of the extracted eluate fractions from ZS. In this case, the pH was determined by the test material itself. Each leachant exchange was followed by a rapid neutralization of the acid by the waste. The eluate fractions from the LCS waste samples had a diametrically different reaction. The pH values of the extracts from the LCS pH7 sample ranged from 7.18 to 8.44, and from the LCS pH4 sample from 3.68 to 4.46. In the latter case, the effect of the interaction of the leaching liquid with a reduced pH on the obtained pH values of the eluates, which stabilized around pH 4, can be observed.

For the ZS waste, the EC values ranged from 19.5–37.4 mS/cm (ZS pH7) to 15.1–26.2 mS/cm (ZS pH4). High conductivity indicates high mobility of ions from monoliths to aqueous phase. The trends of conductivity changes are similar to the trends of pH changes. The EC values of the LCS eluates were much lower than those of ZS and ranged from 18.0 to 46.1 µS/cm. For the LCS waste, higher conductivity was determined for eluates from the sample filles with liquid at pH 4 (LCS pH4 sample).

**Figure 3:**
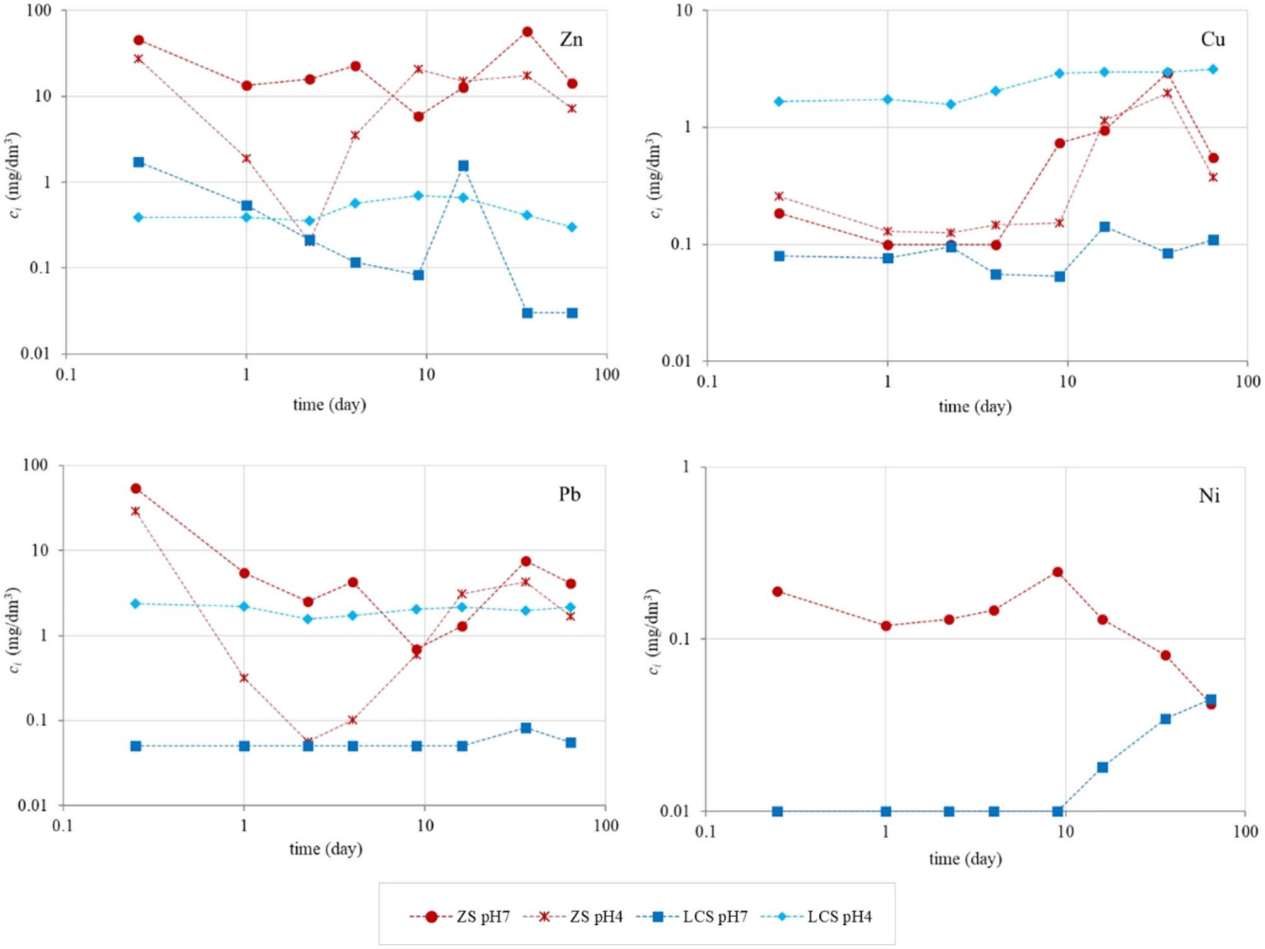
Concentration of Zn, Cu, Pb and Ni (in mg/dm^3^) obtained in the tank test as a function of time.

Figure 3 shows the concentrations of Zn, Cu, Pb, Ni in the eluate fractions obtained from two slags leached with liquid at pH 7 and 4 as a function of time. When the concentration of an element is determined below the limit of quantification, the value of the limit is marked on the graph.

For the analysis of the trend in metal release over time, it was observed that the highest increase in zinc concentration of the ZS pH7 sample occurred in the 7th test period, i.e., after 36 days of the test. Zinc from the ZS pH4 sample was characterized by lower leachability in the 2nd–4th stage of the study, compared to the other eluate fractions. On the other hand, in fractions 5–8, 65% of this element was leached in relation to the total amount leached in the test. Zinc from LCS pH7 sample showed a high concentration already after 0.25 day. Again, a significant intensification of leaching occurred at stage 6 of the test (after 16 days). Following that, another decrease in the release of this element was observed. This may indicate depletion of mobile forms of zinc. The highest leaching of zinc from the sample of LCS pH4 was observed after 9 and 16 days of the study, after which the concentration also decreased.

The leachability of copper from ZS waste was different. During the first 4 days of the test only 5.5% (pH7) and 15% (pH4) were released. The highest concentration of this element was determined at stage 7 for both samples. In the case of LCS pH7 sample the highest concentration of copper was determined after 16 days of testing. Noteworthy is the continuous increasing trend in copper leaching from the LCS pH4 sample, which indicates the availability of this element for leaching even after 64 days of testing.

The highest concentration of lead from ZS pH7 in the amount of 83% was observed already in the first 4 stages of the study. From sample of ZS pH4 this element was released in 75% already after 0.25 day. Equally high levels of lead leaching at the beginning of the test procedure were obtained by Štulović et al.^35^ in the study of sodium lead slag stabilized in concrete using different additives. Lead from LCS pH7 sample leached below the limit of quantification for the first 16 days. Concentrations above the limit were recorded on days 36 and 64 of the study. In contrast, lead from LCS pH4 sample showed similar concentration values throughout the test.

The curve of nickel concentrations from ZS leached with liquid at pH 7 took a different shape than the curves of other heavy metals analyzed for this waste. In the last three fractions of eluates (after 16 days of testing) there was a significant decrease of concentration values. This indicates the depletion of nickel ions available for leaching. In contrast, in the case of nickel from LCS leached with a pH 7 leachant, a significant increase in leaching is observed after 16 days, persisting until the end of the test time.

Table 4 shows the measured cumulative leaching ($\varepsilon_{n}^{*}$) of an element per unit area of waste in contact with the liquid (in mg/m^2^). Measured cumulative leaching ($\varepsilon_{n}^{*}$) is the sum of measured leaching (*E*_i_^*^) obtained in individual eluate fractions extracted during 64 days of testing. When metal concentrations below the limit of quantification are present, the result ($\varepsilon_{n}^{*}$) is given as a range. The uncertainty $\left( {\varepsilon_{n}^{*} } \right)$ was calculated as the root of the sum of the squares of the uncertainties of the leaching results in the individual eluate fractions.

**Table 4:** Measured cumulative leaching ($\varepsilon_{n}^{*}$) of Zn, Cu, Pb, Ni obtained in the tank test (mean ± *u*_*c*_ ($\varepsilon_{n}^{*}$), *n* = 8).

Sample	Measured cumulative leaching ($\varepsilon_{n}^{*}$) (mg/m^2^)			
	Zn	Cu	Pb	Ni
ZS pH7	6630 ± 135	(189–200) ± 11	2834 ± 87	38.5 ± 1.3
ZS pH4	2605 ± 61	120 ± 4	1099 ± 46	< 0.3
LCS pH7	(121–123) ± 5	20 ± 1	(4–13) ± 1	(2.8–4.2) ± 0.2
LCS pH4	151 ± 3	760 ± 24	647 ± 8	< 0.4

The highest zinc release among the test materials analyzed was observed for the ZS waste, as in the other leaching tests. For this waste, generally lower metal concentrations were determined throughout the test cycle when flooded with the pH 4 leachant. At the reduced leachant pH, nickel concentrations below the quantification limit were also determined, while at pH 7 the element leached at 38.5 mg/m^2^. Thus, the effect of acidic environment on the increase of heavy metal cation concentrations from ZS in the monolithic form can be excluded. Although the analytical samples were representative of the entire study material, heavy metals were released at different levels. This may also indicate the heterogeneity of the tested "lumps" of material.

For the LCS waste, on the other hand, much higher leachability of Cu and Pb was recorded at leaching leachant pH 4 than pH 7. In the case of zinc, the concentrations varied over 64 days (Fig. 3), but the total leachability was also higher than at pH 4. For Zn, Cu and Pb, the effect of changing the pH of the leachant on the leachability of selected heavy metals was observed (pH values of the eluates (Fig. 2) remained around 4 throughout the test). Nickel, on the other hand, was an exception, since at pH 4 its release was determined below the limit of quantification throughout the test cycle.

The increased leachability of heavy metals under acidic conditions is most likely related to the occurrence of specific mineral dissolution reactions (especially sulfides) and metal desorption reactions from reactive surfaces, such as iron oxides^36^. Dissolution processes also depend on the mineral phases present in the waste and their chemical composition. Leaching of metals from wastes in the monolithic form can occur by various mechanisms, such as diffusion, dissolution, or leaching from surfaces, which in turn affects the quantity of leachability. Based on the authors' research also published in other papers^37,38^, it was observed that the mechanisms controlling the heavy metals release also depends on duration of leaching process . For some materials, the leaching process may increase after several days of contact with water (see Fig. 3), when the leaching mechanism is changed. This can determine the actual leaching levels over time. The authors emphasize that it is also important to know the maximum amount of the element available for leaching. Only such a comprehensive assessment of the leachability of heavy metals can determine the correct direction of waste management. There are many “application scenarios” of waste materials (e.g. placement in the ground or on its surface, contact with water, carbonation). From a practical point of view, this is important because that the available and used leaching methods as single tests are incomplete for a proper waste assessment. It seems that only a comprehensive approach, taking into account various methods simulating various factors, will show the actual level of leaching of heavy metals. However, in this process, it is necessary to develop a multi-criteria decision-making method in the selection of the leaching method. It was also concluded that the contaminant leachability test procedure based on EN 12457:2002 is insufficient in assessing the potential environmental impact of the waste. It does not fully reflect the degree of danger to the environment that may be posed by waste during its storage, reuse and introduction to the environment, e.g. in the form of aggregates or reclamation material. The method also does not fully reflect the conditions in which waste materials may be found in the natural environment. As a result of the influence of atmospheric factors, chemical weathering processes, changes in pH of the surrounding environment or oxidizing and reducing conditions, the level of leaching contaminants may increase and thus the permissible concentration limits for a given waste type (inert, hazardous and other than inert and hazardous) may be exceeded.

## Conclusion

One of the purposes of laboratory leachability tests may be to predict the long-term release of constituents from waste materials. It is not possible to obtain test results in a realistic time frame under laboratory conditions. Simulating long-term release scenarios can be done by temporarily increasing the leachant flow rate through the column or increasing the leachant volume in batch tests. Both procedures can simulate exposure of the material to natural factors, primarily atmospheric precipitation, for extended periods of time.

Column (percolation) tests conducted until at least L/S = 10 dm^3^/kg can be used to assess the long-term leaching behaviour of heavy metals. Batch tests conducted at L/S = 10 dm^3^/kg, on the other hand, are a good tool to quickly assess wastes for the content of mobile forms of heavy metals. Studies have generally obtained higher metal leachability throughout the percolation test procedure than the batch test.

Long-term leaching behaviour of heavy metals from wastes subjected to long-term exposure to extreme environmental factors, such as low pH, can be determined by evaluating the maximum amount of element available for leaching. It was observed that the high L/S = 50 dm^3^/kg ratio, in addition to the low pH of the leaching liquid and the refinement of the waste to < 0.125 mm, could significantly affect the release of Zn, Cu, Pb and Ni at the highest levels among all test methods.

The observation of changes in the leaching of heavy metals from monolithic waste over a long period of time (64 days) indicated an ambiguous trend in the intensity of metal release depending on the study period and the material tested. Intensification of leaching of some metals, such as Cu or Ni from lump copper slag (pH4) occurred only after 16 days of contact between the waste and the leachant. The leaching process over a long period of time was also dependent on pH of eluent. Monolithic lump copper slags released more portions of heavy metals at pH 4 of the leachant. Such a relationship was not observed for zinc slags.

The obtained results clearly indicate that it is difficult to determine the direction of waste management in connection with the leaching prediction of heavy metals. There are many chemical and physical factors affecting the leachability of heavy metals. The assessment of heavy metals release should be conducted with multi-criteria analysis taking into account various application scenarios of waste.

## Data Availability

All research results are included in the paper. The datasets available from the corresponding author on reasonable request.
